# Myeloperoxidase Antineutrophil Cytoplasmic Antibody (MPO-ANCA)-Associated Usual Interstitial Pneumonia: Pathogenic Mechanisms, Diagnostic Challenges, and Clinical Implications

**DOI:** 10.7759/cureus.108728

**Published:** 2026-05-12

**Authors:** Stephanie Johanna Chon Pineda, Mariana Baron-Anaya, Valeria Arroyo-Cruz, Ana-Paola Puente-Gutu, Alvaro Paul Calvo-Cantú, Dante Arroyo-Cruz, María Fernanda Aguirre Quintero, Alfonso Sandoval, Ashley Guzmán Velázquez, José Emiliano González Flores

**Affiliations:** 1 School of Medicine and Health Sciences, Tecnológico de Monterrey, Campus Ciudad de México, Mexico City, MEX; 2 Faculty of Health Sciences School of Medicine, Universidad Panamericana, Mexico City, MEX; 3 School of Medicine, Universidad Regional del Sureste, Oaxaca, MEX; 4 Department of Internal Medicine, Instituto Mexicano del Seguro Social (IMSS) Hospital General Regional No. 23, Baja California, MEX; 5 Medical Research and Academic Mentorship, ShareTheMed Research Group, Mexico City, MEX; 6 Faculty of Public Health, Universidad Anáhuac México, Mexico City, MEX

**Keywords:** anca-associated vasculitis, interstitial lung disease, microscopic polyangiitis, mpo-anca, pulmonary fibrosis, usual interstitial pneumonia

## Abstract

Usual interstitial pneumonia (UIP) has traditionally been regarded as synonymous with idiopathic pulmonary fibrosis (IPF) in the absence of an identifiable cause. However, emerging evidence demonstrates that a subset of patients with a UIP pattern exhibit myeloperoxidase antineutrophil cytoplasmic antibodies (MPO-ANCAs), suggesting a distinct and evolving clinical phenotype at the intersection of fibrotic lung disease and systemic autoimmunity. This structured narrative review aims to synthesize current evidence regarding the clinical, diagnostic, and therapeutic implications of MPO-ANCA-associated UIP. Preferred Reporting Items for Systematic Reviews and Meta-Analyses-like methodology was employed to identify and select relevant studies from PubMed (MEDLINE) and Google Scholar published between 2020 and 2026. After screening and eligibility assessment, 18 studies were included in the qualitative synthesis. Data were extracted on clinical presentation, radiological features, serological findings, therapeutic strategies, and outcomes. The available evidence indicates that pulmonary fibrosis may precede or coexist with ANCA-associated vasculitis, particularly microscopic polyangiitis, supporting the concept of UIP as a potential early or lung-dominant manifestation of systemic disease. This phenotype is characterized by a dual trajectory, combining progressive fibrosis with the risk of severe vasculitic complications such as diffuse alveolar hemorrhage. Radiological findings alone are insufficient for differentiation, highlighting the importance of MPO-ANCA testing for early phenotyping. Therapeutic management remains challenging due to the lack of standardized guidelines, requiring individualized and multidisciplinary approaches that balance antifibrotic and immunosuppressive strategies. MPO-ANCA-associated UIP should be recognized as a distinct and dynamic clinical entity with significant diagnostic and prognostic implications. Early identification and structured follow-up are essential to optimize outcomes and guide timely therapeutic interventions.

## Introduction and background

Usual interstitial pneumonia (UIP) is a radiologic and histopathologic pattern characterized by heterogeneous pulmonary fibrosis with basal and subpleural predominance, architectural distortion, and honeycombing [1]. Although historically regarded as synonymous with idiopathic pulmonary fibrosis (IPF) in the absence of an identifiable cause, UIP is now recognized as a non-specific pattern that may arise in a variety of clinical contexts [2]. Accordingly, while IPF is defined by the presence of a UIP pattern after exclusion of known etiologies, the identification of UIP does not, by itself, establish a diagnosis of IPF [2,3].

Accumulating evidence indicates that a subset of patients with a UIP pattern harbor antineutrophil cytoplasmic antibodies directed against myeloperoxidase (MPO-ANCA), a phenomenon described for several decades but increasingly recognized for its clinical relevance [1]. This association challenges the assumption that UIP is invariably idiopathic and highlights the need for a more nuanced diagnostic framework integrating serological and clinical features [1-3].

The clinical relevance of MPO-ANCA-associated UIP lies in its dynamic and heterogeneous disease course, which extends beyond a purely fibrotic process [4]. Pulmonary fibrosis may precede the onset of ANCA-associated systemic vasculitis, particularly microscopic polyangiitis, and delayed seroconversion to MPO-ANCA positivity has been reported during follow-up in patients initially classified as seronegative [4-6]. Moreover, the coexistence of UIP and MPO-ANCA positivity has been associated with worse clinical outcomes compared with isolated interstitial lung disease or vasculitis without fibrosing involvement, underscoring the importance of early recognition, longitudinal surveillance, and timely therapeutic decision-making [7-9].

Despite increasing recognition of this entity, substantial gaps in the literature persist. Although well-established international guidelines exist for the management of ANCA-associated vasculitis and IPF as separate entities, no specific recommendations address the combined phenotype of MPO-ANCA-associated UIP. Consequently, therapeutic decision-making remains challenging, not due to the absence of guidance, but because of uncertainty regarding treatment response and the need to individualize management strategies.

In this context, clinicians must balance the use of immunosuppressive therapies typically employed in ANCA-associated vasculitis with antifibrotic agents indicated for fibrotic lung disease, or consider a combination of both depending on the predominant disease component. Furthermore, ongoing debate persists as to whether MPO-ANCA-associated UIP represents a pulmonary-limited manifestation within the spectrum of ANCA-associated vasculitis or a distinct clinical phenotype with overlapping fibrotic and autoimmune features.

In this context, the present review aims to highlight the diagnostic and therapeutic challenges associated with MPO-ANCA-associated UIP and to emphasize its clinical relevance as a distinct and evolving phenotype that challenges conventional IPF-based diagnostic and management paradigms.

## Review

Methodology

Study Design

This study was conducted as a structured narrative review addressing UIP associated with MPO-ANCA. A Preferred Reporting Items for Systematic Reviews and Meta-Analyses (PRISMA)-informed approach was followed for the identification, screening, and selection of relevant studies, ensuring a systematic and transparent process throughout the review. Electronic databases were explored using predefined search strategies, and studies were evaluated according to eligibility criteria established before data extraction. Given the heterogeneity in study designs, patient populations, and reported outcomes, quantitative synthesis was deemed inappropriate, and no meta-analysis was performed.

Search Strategy

The search was conducted using PubMed (MEDLINE) and Google Scholar for studies published between 2020 and 2026, with the last search performed on February 9, 2026. The search strategy was designed to maximize sensitivity for both fibrotic and vasculitic phenotypes.

A combination of MeSH terms and free-text keywords was applied using Boolean operators. The PubMed search string was (“Usual Interstitial Pneumonia” OR “UIP” OR “Idiopathic Pulmonary Fibrosis” OR “IPF” OR “Pulmonary Fibrosis”) AND (“ANCA-associated vasculitis” OR “ANCA” OR “MPO-ANCA” OR “Myeloperoxidase” OR “anti-myeloperoxidase” OR “p-ANCA” OR “Microscopic Polyangiitis”) AND (“Interstitial Lung Disease” OR “ILD” OR “Interstitial Pneumonia”), applying filters for humans, adults, and MEDLINE-indexed articles. The Google Scholar search string was “Usual interstitial pneumonia” AND “MPO-ANCA.”

The search was limited to selected databases, excluding grey literature and studies without full-text availability, which may have limited comprehensiveness but remained consistent with the structured narrative design.

Inclusion Criteria

Inclusion criteria were defined before study selection in a structured manner. Only articles with full-text availability were considered. Eligible publication types included clinically relevant studies such as case reports, case series, cohort studies, and narrative or systematic reviews. The population of interest was limited to adult human subjects. Studies addressing UIP in association with MPO-ANCA, including diagnostic approaches and pathophysiological aspects, were considered. Articles were required to report relevant clinical, radiological, serological, or pathological outcomes. Only studies published in English or Spanish were included.

Exclusion Criteria

Exclusion criteria were established to ensure the inclusion of clinically relevant studies aligned with the scope of the review. Editorials, letters to the editor, and opinion pieces without original clinical data were excluded, as were isolated descriptions of surgical techniques without clinical outcome assessment. Studies in animal models or preclinical research were excluded, as were those that did not report relevant clinical outcomes, including mortality, disease progression, therapeutic response, or complications. Articles focused on conditions unrelated to MPO-ANCA-associated UIP or studies addressing pulmonary fibrosis without ANCA/MPO assessment were also excluded. Furthermore, studies in pediatric populations (under 18 years of age) and articles published in languages other than English or Spanish were excluded.

Study Selection Process

**Figure 1:**
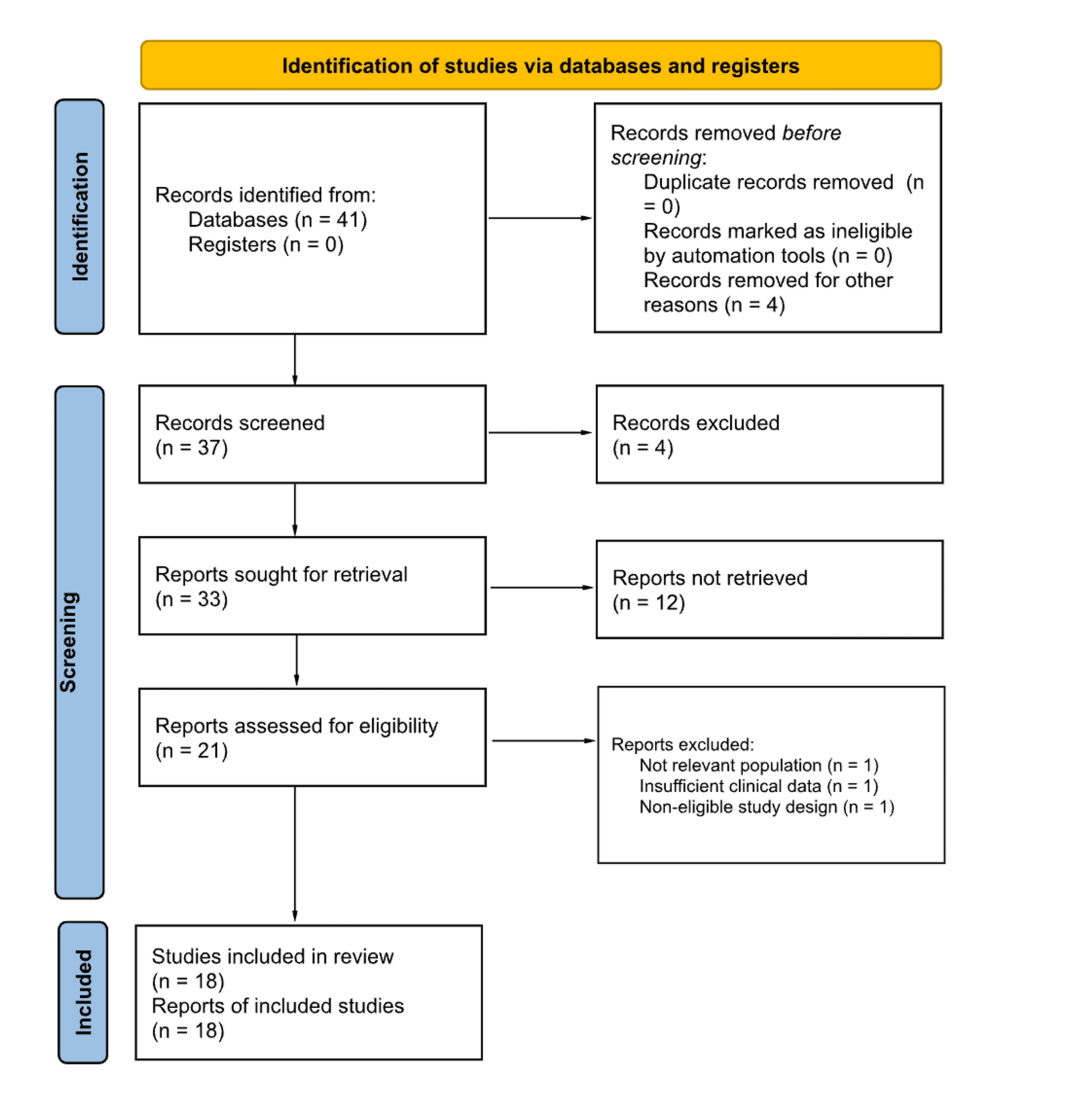
Preferred Reporting Items for Systematic Reviews and Meta-Analyses-informed flow diagram of study selection. Preferred Reporting Items for Systematic Reviews and Meta-Analyses-informed flow diagram illustrating the process of study identification, screening, eligibility assessment, and inclusion.

The study selection process followed a PRISMA-informed approach to ensure a structured and transparent identification and screening of relevant studies, as illustrated in Figure 1. A total of 41 records were initially identified through database searching. Before screening, four records were removed for other reasons, resulting in 37 records undergoing title and abstract screening, of which four were excluded. Subsequently, 33 reports were sought for retrieval, of which 12 were not accessible in full text. A total of 21 studies were assessed for eligibility. Of these, three reports were excluded due to a non-relevant population (n = 1), insufficient clinical data (n = 1), and a non-eligible study design (n = 1). Finally, 18 studies met the inclusion criteria and were included in the qualitative synthesis.

Data Extraction

Data extraction was performed using a predefined structured form to systematically organize relevant information from each included study, ensuring consistency and facilitating analysis. Variables were defined a priori based on the review objectives and included population characteristics (age, sex, and sample size), clinical features (presence of UIP and pulmonary and systemic manifestations), and diagnostic parameters (radiological findings, MPO-ANCA serology, and lung biopsy when available). Data related to therapeutic approaches (immunosuppressive therapy, antifibrotic agents, and other interventions) and clinical outcomes (disease progression, mortality, and complications) were also collected. All data were extracted directly from full-text articles and recorded in a standardized format to reduce variability in interpretation and ensure consistency across studies.

Quality Assessment

Given the narrative design and heterogeneity of the included studies, a formal risk of bias assessment using standardized tools was not performed. However, key methodological characteristics, including study design, sample size, presence of comparator groups, and duration of follow-up, were systematically extracted to contextualize the strength and limitations of the available evidence.

Synthesis Strategy

Due to the heterogeneity in study designs, patient populations, and reported outcomes, a narrative synthesis was performed. Findings were grouped into predefined thematic domains based on the data extraction framework, including general study characteristics, temporal relationship between interstitial lung disease and ANCA-associated vasculitis, radiological and functional features, and therapeutic strategies with clinical outcomes. No statistical pooling or meta-analysis was conducted.

Literature review

The general characteristics of the included studies are summarized in Table 1, highlighting the predominance of retrospective cohort designs, UIP patterns on high-resolution computed tomography (HRCT, and variable rates of vasculitic involvement across populations [4,5,7,8,10-23].

**Table 1:** General characteristics of included studies. NR = not reported; HRCT = high-resolution computed tomography; UIP = usual interstitial pneumonia; NSIP = non-specific interstitial pneumonia; ANCA = antineutrophil cytoplasmic antibodies; MPO-ANCA = myeloperoxidase antineutrophil cytoplasmic antibodies

Author (year)	Country	Study design	Sample size (N)	Mean/Median age (years)	Male (%)	MPO-ANCA positivity (%)	Predominant HRCT pattern	Vasculitis present (%)
Sun et al., 2025 [4]	NR	Retrospective cohort	NR	60	47.5	100	NSIP	NR
Maillet et al., 2020 [5]	France/Belgium	Multicenter retrospective cohort	34	68	61	89	UIP	100
Yamakawa et al., 2021 [7]	Japan	Retrospective cohort	9	75	50.8	100	Probable UIP	36
Wurmann et al., 2020 [8]	Chile	Retrospective cohort	101	65	NR	94	UIP	100
Lee et al., 2024 [10]	South Korea	Retrospective cohort	NR	68	63	100	UIP	NR
Casal Moura et al., 2025 [11]	USA	Retrospective cohort	143	73	31	74.8	UIP	100
Song et al., 2024 [12]	China	Case-control cohort	NR	71	61	90	UIP	100
Kaya et al., 2025 [13]	Turkey	Cross-sectional study	83	47.6	55.4	NR	UIP	100
Sakamoto et al., 2023 [14]	Japan	Longitudinal cohort	NR	71	50	100	UIP	NR
Zhu et al., 2024 [15]	China	Retrospective cohort	NR	60	51.9	70.4	UIP	No
Matsuda et al., 2024 [16]	Japan	Multicenter cohort	89	75	NR	98	UIP	NR
Yamaguchi et al., 2023 [17]	Japan	Retrospective cohort	NR	73	83	100	NR	Yes
Chalkia et al., 2025 [18]	Greece	Case report	1	65	NR	100	UIP	100
Nakayama et al., 2023 [19]	Japan	Case report	1	70	NR	NR	UIP (suggestive)	NR
Shimamura et al., 2025 [20]	Japan	Retrospective cohort	19	71	54.4	100	UIP	29.8
Sebastiani et al., 2021 [21]	Italy/Spain	Multicenter cohort	30	69	46	100	UIP	17
Yang et al., 2022 [22]	China	Retrospective cohort	72	69	59.7	100	UIP	NR
Doliner et al., 2022 [23]	USA	Multicenter cohort	91	67	46	93	UIP	NR

Clinical Definition and Diagnostic Relevance of UIP With MPO-ANCA Positivity

UIP associated with MPO-ANCA represents a clinically relevant autoimmune phenotype that challenges the traditional interpretation of fibrotic lung disease as idiopathic. The UIP pattern, characterized by peripheral reticulation, traction bronchiectasis, and honeycombing, is classically associated with IPF; however, it is now recognized as a non-specific radiologic and histopathologic pattern that may occur across multiple etiologies [2].

In this context, the presence of MPO-ANCA positivity introduces a distinct pathophysiological dimension, suggesting that, rather than a purely fibrotic disorder, this condition may reflect an early or lung-dominant manifestation within the spectrum of ANCA-associated vasculitis [5]. Clinically, the identification of MPO-ANCA has direct implications for disease classification, as it may prompt the reclassification of patients initially considered to have idiopathic disease into a phenotype at risk for systemic vasculitic evolution.

This distinction is clinically meaningful, as it redefines a seemingly isolated pulmonary condition into a systemic disease continuum, influencing diagnostic evaluation, longitudinal surveillance, therapeutic decision-making, and prognostic stratification [8].

Epidemiology and Demographic Profile

The epidemiology of UIP associated with MPO-ANCA demonstrates distinct demographic patterns. The prevalence of MPO-ANCA positivity among patients initially diagnosed with UIP or IPF is estimated to range between 4% and 14% [3].

This condition predominantly affects older adults, with a reported age of onset between 65 and 75 years [6]. Sex distribution appears relatively balanced, although a slight female predominance has been described in certain cohorts [7].

From a geographical perspective, Asian populations have reported higher rates of pulmonary involvement as the initial manifestation of ANCA-associated vasculitis compared to Western cohorts, suggesting potential contributions from genetic susceptibility and environmental factors [6].

Clinically, these epidemiological patterns highlight the importance of maintaining a low threshold for MPO-ANCA testing in patients presenting with fibrotic interstitial lung disease, even in cases that initially appear consistent with idiopathic disease [17].

Temporal Relationship Between UIP and Systemic Vasculitis

The temporal relationship between fibrotic interstitial lung disease and systemic vasculitis is highly variable. Accumulating evidence suggests that pulmonary fibrosis may represent a prolonged preclinical phase of disease. In a substantial proportion of patients, the diagnosis of a UIP pattern precedes the clinical onset of microscopic polyangiitis (MPA) by months or even years [5,8].

Available studies indicate that the interval between the initial detection of fibrosis and the development of overt systemic vasculitis frequently occurs within the first two years following diagnosis [9]. Furthermore, longitudinal data demonstrate that approximately 10% to 25% of patients initially classified as having ANCA-positive idiopathic interstitial pneumonia eventually develop systemic manifestations of vasculitis [10].

From a pathophysiological perspective, this temporal sequence suggests that the lung parenchyma may serve as an initial site of immune dysregulation. Accordingly, fibrotic interstitial lung disease should not be regarded solely as a late complication, but rather as a potential early sentinel event requiring structured longitudinal surveillance and clinical vigilance for systemic progression [9,11,16].

As summarized in Table 2, interstitial lung disease frequently precedes the onset of systemic vasculitis, often by several months or years, supporting the concept of pulmonary fibrosis as a potential preclinical phase of ANCA-associated disease.

**Table 2:** Temporal relationship between ILD and ANCA-associated vasculitis. NR = not reported; ILD = interstitial lung disease; AAV = antineutrophil cytoplasmic antibody-associated vasculitis; ANCA = antineutrophil cytoplasmic antibodies; AAV-ILD = ANCA-associated vasculitis with interstitial lung disease; ANCA-ILD = ANCA-positive interstitial lung disease; PLV = pulmonary-limited vasculitis; DAH = diffuse alveolar hemorrhage

Author (year)	Study population	ILD preceding AAV (%)	AAV preceding ILD (%)	Simultaneous diagnosis (%)	Time interval	Renal involvement (%)	DAH (%)
Sun et al., 2025 [4]	ANCA-ILD	NR	NR	NR	NR	NR	NR
Maillet et al., 2020 [5]	AAV-ILD	37	NR	70	NR	26	18
Yamakawa et al., 2021 [7]	ANCA-ILD	NR	NR	NR	NR	NR	9.8
Wurmann et al., 2020 [8]	AAV-ILD	58.8	NR	NR	0.5–14 years	47	NR
Lee et al., 2024 [10]	ANCA-ILD	NR	NR	NR	1–83 months	75	NR
Casal et al., 2025 [11]	AAV-ILD	44.1	NR	68.2	NR	NR	NR
Song et al., 2024 [12]	AAV-ILD	NR	NR	68.3	NR	NR	10
Kaya et al., 2025 [13]	AAV-ILD	NR	NR	63.9	NR	NR	NR
Sakamoto et al., 2023 [14]	ANCA-ILD	NR	NR	NR	NR	NR	NR
Zhu et al., 2024 [15]	ANCA-ILD	NR	NR	NR	NR	NR	NR
Matsuda et al., 2024 [16]	AAV-ILD	NR	NR	2.2	NR	NR	NR
Yamaguchi et al., 2023 [17]	ANCA-ILD	NR	NR	NR	NR	89	11
Chalkia et al., 2025 [18]	Case report	100	NR	100	NR	NR	100
Nakayama et al., 2023 [19]	Case report	NR	NR	NR	27 months	NR	NR
Shimamura et al., 2025 [20]	AAV-ILD + PLV	NR	NR	NR	NR	NR	NR
Sebastiani et al., 2021 [21]	ANCA-ILD	100	NR	NR	27 months	NR	NR
Yang et al., 2022 [22]	AAV-ILD	NR	NR	NR	4.9 years	75	NR
Doliner et al., 2022 [23]	AAV-ILD	85	NR	NR	3.3 years	57	13

Radiological and Diagnostic Features

The diagnostic approach to MPO-ANCA-associated lung disease requires the integration of HRCT findings with targeted serological evaluation, as radiological features alone are insufficient to reliably distinguish it from idiopathic disease. HRCT typically demonstrates a definite or probable UIP pattern, characterized by basilar-predominant reticular opacities, traction bronchiectasis, and honeycombing [5].

Although these imaging features are classically associated with IPF, they are not disease-specific, and the presence of MPO-ANCA represents a critical element for accurate phenotypic classification. In this context, mixed radiological patterns, such as the coexistence of upper-lobe emphysema and lower-lobe fibrosis (combined pulmonary fibrosis and emphysema, CPFE), have been reported with increased frequency and are associated with worse clinical outcomes [11].

Given the inability of imaging to predict the underlying autoimmune trajectory, MPO-ANCA testing should be considered in patients presenting with fibrotic interstitial lung disease, particularly when clinical, laboratory, or radiological features are atypical for isolated disease. In most cases, the combination of HRCT findings and serological markers is sufficient to establish the diagnosis, and lung biopsy is rarely required, being reserved for cases with diagnostic uncertainty [14,18,20].

This integrated diagnostic approach enables early phenotyping and reduces the risk of misclassifying a potentially evolving systemic vasculitis as a purely idiopathic fibrotic disorder, thereby facilitating appropriate clinical surveillance and management [10].

Clinical Manifestations and Extrapulmonary Involvement

The clinical presentation of MPO-ANCA-associated UIP is characterized by a dynamic and heterogeneous course, often evolving from a pulmonary-limited phase to systemic vasculitic involvement. In the early stage, symptoms are typically insidious and dominated by progressive exertional dyspnea and chronic dry cough [10].

As the disease progresses, patients may develop systemic manifestations including fatigue, myalgias, weight loss, and, most notably, rapidly progressive glomerulonephritis, reflecting transition to overt ANCA-associated vasculitis [12]. Diffuse alveolar hemorrhage (DAH) represents one of the most severe complications, often occurring abruptly and associated with significant mortality [13].

Importantly, while UIP with MPO-ANCA positivity may initially present as a lung-dominant phenotype, the most common clinical manifestation of MPO-ANCA-associated vasculitis in broader clinical practice remains renal involvement, frequently in the context of a pulmonary-renal syndrome. This distinction highlights the potential for specialty-related diagnostic bias, in which pulmonology-focused cohorts may overrepresent fibrotic presentations [16,20,21].

Clinically, this underscores the importance of maintaining a high index of suspicion for systemic involvement. Subtle findings such as unexplained anemia, sudden declines in hemoglobin, or new-onset hematuria may represent early indicators of vasculitic progression. Recognition of these features is critical, as it necessitates a shift from predominantly antifibrotic or supportive management toward timely initiation of immunosuppressive therapy [20,21].

Prognostic Implications and Clinical Outcomes

The coexistence of UIP and MPO-ANCA is associated with unfavorable outcomes, largely driven by the extent and progression of fibrotic lung disease rather than vasculitic manifestations alone [5]. However, prognosis remains heterogeneous and is strongly influenced by radiological patterns, with non-UIP forms generally associated with a more favorable clinical course compared to UIP patterns [7].

Multiple factors contribute to adverse outcomes, including acute complications, chronic disease progression, and relapse. Acute events such as exacerbations of interstitial lung disease and DAH are consistently associated with reduced survival [12]. Chronic progression of fibrosis is observed in a substantial proportion of ANCA-associated interstitial lung disease (ANCA-ILD) patients, particularly among older individuals, those with elevated surfactant protein-D (SP-D) levels, lower baseline forced vital capacity, or persistent functional decline despite treatment [14,15]. Given its dynamic nature, ANCA-positive interstitial pneumonia may evolve into overt systemic vasculitis over time, particularly in patients with anti-MPO positivity or concomitant autoimmune markers such as rheumatoid factor. However, quantitative anti-MPO titers were not consistently reported across the included studies, and assessment methods varied across institutional serological platforms, most commonly enzyme-linked immunosorbent assay or other immunoassay-based approaches. Therefore, a comparative analysis of anti-MPO titers was not feasible in this review. This evolving trajectory further complicates prognosis and challenges the application of conventional IPF-based management strategies in this population [10].

Additionally, relapse following immunosuppressive therapy occurs more frequently in patients with a UIP pattern and elevated biomarkers such as KL-6 and SP-D, and is associated with increased all-cause and respiratory mortality [16,17].

Therapeutic Approaches and Current Uncertainties

Although UIP with MPO-ANCA positivity should not be automatically classified as IPF, well-established international guidelines exist for the management of ANCA-associated vasculitis and fibrotic interstitial lung disease as separate entities. However, no specific recommendations currently address this combined phenotype, making therapeutic decision-making particularly challenging.

Management, therefore, requires an individualized, multidisciplinary approach that integrates both fibrotic and vasculitic components of the disease. Across available cohorts, corticosteroids are commonly used in combination with immunosuppressive agents such as cyclophosphamide, while rituximab is typically reserved for selected cases or combination strategies [7,8].

Treatment should be guided by the predominant disease phenotype. Patients with fibrotic-predominant UIP patterns may derive greater benefit from antifibrotic therapies, whereas those with non-UIP or inflammatory-dominant patterns tend to respond more favorably to immunosuppressive treatment [11]. This distinction is clinically relevant, as the use of immunosuppressive therapy in predominantly fibrotic disease may expose patients to treatment-related toxicity without clear pulmonary benefit [5].

Combination therapy may be considered in cases where both fibrotic and vasculitic mechanisms coexist; however, current evidence remains limited, largely non-comparative, and insufficient to support definitive treatment algorithms.

Emerging therapies such as avacopan, a selective complement C5a receptor inhibitor, may offer a steroid-sparing alternative in severe or complex ANCA-associated vasculitis with interstitial lung disease, although evidence is currently restricted to highly selected populations [18].

Overall, the management of MPO-ANCA-associated UIP remains guided by clinical judgment, requiring careful phenotypic assessment and close multidisciplinary collaboration. As summarized in Table 3, therapeutic strategies remain highly heterogeneous across studies, with frequent use of corticosteroids and immunosuppressive agents, while antifibrotic therapies are variably incorporated depending on the predominant disease phenotype.

**Table 3:** Treatment strategies and clinical outcomes. NR = not reported; ILD = interstitial lung disease; AAV = antineutrophil cytoplasmic antibody-associated vasculitis; ANCA = antineutrophil cytoplasmic antibodies; AAV-ILD = ANCA-associated vasculitis with interstitial lung disease; ANCA-ILD = ANCA-positive interstitial lung disease; PLV = pulmonary-limited vasculitis; DAH = diffuse alveolar hemorrhage

Author (year)	Corticosteroids (%)	Cyclophosphamide (%)	Rituximab (%)	Other immunosuppressants	Antifibrotics	Acute exacerbation (%)	Mortality (%)	Survival data
Sun et al., 2025 [4]	85.2	23	NR	4.9%	Yes	9.8	18	NR
Maillet et al., 2020 [5]	100	79	42 (maintenance), 11 (induction)	Azathioprine 47%, methotrexate 3%, mycophenolate 16%	Yes	33	HR = 2.73 (1.44–5.19)	Reduced survival in UIP-AAV
Yamakawa et al., 2021 [7]	NR	NR	NR	Anti-inflammatory agents 57.4%	Yes	6.6	14.8	5-year mortality (UIP): 61.1%
Wurmann et al., 2020 [8]	100	82.4	17.6	Azathioprine 100%	NR	NR	30	Kaplan-Meier only
Lee et al., 2024 [10]	57.8	NR	NR	Pirfenidone 43.5%, nintedanib 1.9%, MMF 16.9%, azathioprine 8.4%, cyclosporine 4.5%	Yes	NR	19.5	33.3%
Casal et al., 2025 [11]	15.4	58	NR	Azathioprine 34.3%, MMF 18.9%	NR	NR	45.5	Best survival in UIP (1–10 years)
Song et al., 2024 [12]	68.3	NR	8.3	Combination therapy 50%	Yes	6.7	14.8	Not specified
Kaya et al., 2025 [13]	100	57	45.8	Azathioprine 49.4%, methotrexate 25.3%, MMF 18.1%	NR	NR	NR	NR
Sakamoto et al., 2023 [14]	100 (MPA-ILD), 58.8 (ANCA-IP)	NR	NR	NR	Yes	NR	NR	PPF: median 2046 days
Zhu et al., 2024 [15]	88.9	25.9	NR	3.7%	No	24.8	22.2	77.8%
Matsuda et al., 2024 [16]	100	NR	Not specified	NR	NR	75	66	56 patients
Yamaguchi et al., 2023 [17]	100	23	7	NR	Yes	11	9	34%
Chalkia et al., 2025 [18]	100	100	100	Avacopan 100%	No	NR	0	100% survival at 6 months
Nakayama et al., 2023 [19]	Yes	NR	NR	NR	Yes (GLPG169 trial)	NR	No	Alive
Shimamura et al., 2025 [20]	68.4	NR	NR	Not specified (37.7%)	NR	NR	30.7	UIP associated with reduced survival
Sebastiani et al., 2021 [21]	64.1	7.5	5.7	20.7%	Yes	22.4	NR	Deaths: IIP 13, AAV 3
Yang et al., 2022 [22]	96.1	NR	0	66.5%	NR	NR	HR 9.94	Median = 39.1 months
Doliner et al., 2022 [23]	NR	NR	NR	Not specified	Yes	7	NR	Mortality: ILD 38% vs, 25%

Clinical Follow-Up and Implications for Practice

In patients with interstitial lung disease, particularly those exhibiting a UIP pattern, diagnostic evaluation should extend beyond IPF, as a subset of cases may reflect an underlying or evolving autoimmune process [7]. This is supported by observations in MPA-associated interstitial lung disease, where patients may present with elevated inflammatory markers such as C-reactive protein, impaired renal function, anemia, and characteristic HRCT features including irregular cystic changes within a UIP pattern, often accompanied by extrapulmonary manifestations [8,12].

In this context, MPO-ANCA testing should be considered in patients with UIP-pattern interstitial lung disease, particularly when clinical, laboratory, or radiological features are atypical for isolated fibrotic disease. Additional findings, such as rheumatoid factor positivity, bronchoalveolar lavage eosinophilia, or low-attenuation areas on imaging, may further identify individuals at risk of subsequent vasculitic evolution, supporting a broader serological evaluation [10,19].

Even in the absence of overt systemic vasculitis, MPO-ANCA seropositivity warrants close clinical and radiological surveillance, given its association with increased morbidity and mortality. Specific imaging features, such as anterior upper-lobe honeycomb-like lesions, may help identify patients at higher risk of adverse outcomes [7,20].

Follow-up should include regular clinical assessment, pulmonary function testing, and serial laboratory, serological, and radiological monitoring to detect early signs of disease progression or systemic involvement [10,21]. This approach extends beyond conventional IPF follow-up paradigms and reflects the need for a more dynamic, phenotype-oriented model of care.

Evidence Gaps and Future Directions

The current body of literature on UIP associated with MPO-ANCA-positive interstitial lung disease remains limited. Most available evidence derives from retrospective observational studies and isolated case reports, often involving small or highly selected populations, which restricts generalizability and limits robust comparisons across clinical outcomes and therapeutic strategies.

A major challenge lies in diagnostic inconsistency, as this condition is variably described across studies using terms such as ANCA-associated vasculitis-interstitial lung disease, MPA-associated-interstitial lung disease, pulmonary-limited vasculitis, MPO-ANCA-positive interstitial lung disease, or ANCA-associated idiopathic interstitial pneumonia. These heterogeneous definitions, frequently combined with variable HRCT-based classifications, hinder the development of uniform diagnostic criteria and complicate the establishment of standardized treatment approaches.

Prospective, multicenter registries incorporating longitudinal serological, functional, and radiological data are essential to better characterize disease evolution and treatment response over time. In parallel, the continued reporting of well-characterized cohorts and cases remains important to identify patterns of delayed vasculitic progression and long-term outcomes [13].

Overall, UIP associated with MPO-ANCA-positive interstitial lung disease should be regarded as a dynamic and evolving clinical phenotype. Advancing its understanding will require standardized definitions, improved phenotypic classification, and collaborative research efforts aimed at developing evidence-based diagnostic and therapeutic frameworks.

Discussion

UIP associated with MPO-ANCA should not be interpreted solely within the framework of IPF, but rather as part of a dynamic clinicopathological continuum bridging fibrotic lung disease and systemic autoimmunity [2,3,5,10,21]. Although the UIP pattern is classically associated with IPF, it is now recognized as a non-specific radiologic and histopathologic pattern that may arise in multiple clinical contexts [2,3]. In this setting, the presence of MPO-ANCA introduces a distinct clinical dimension, suggesting the possibility of an evolving ANCA-associated vasculitis phenotype [3,5,10,21].

This distinction is not merely semantic but clinically meaningful, as it may reframe a seemingly isolated fibrotic disorder into a condition with potential systemic implications, thereby influencing diagnostic evaluation, longitudinal surveillance, and therapeutic decision-making [3,10,21].

The available evidence suggests that pulmonary fibrosis may precede, coincide with, or follow the onset of systemic vasculitis, supporting the concept of a dynamic temporal relationship between interstitial lung disease and ANCA-associated vasculitis [5,8,10,11,21-23]. In this context, UIP may represent an early or lung-dominant manifestation within the spectrum of ANCA-associated disease, rather than a purely coincidental association [3,5,10,21].

Reported rates of seroconversion and progression to MPA further support this interpretation, although the proportion of patients who develop overt systemic disease remains variable across studies [1,6,10,19,21]. From a pathophysiological perspective, this temporal sequence raises the possibility that the lung parenchyma may serve as an initial site of immune dysregulation [3,21].

These observations challenge the traditional view of fibrosis as a purely terminal or isolated process and instead support a model in which fibrotic lung disease may, in selected cases, represent an early stage within a broader systemic disease continuum [3,10,21].

Clinically, this evolving phenotype is characterized by a dual trajectory in which progressive fibrotic lung disease coexists with the risk of acute vasculitic complications [5,8,12,13,17,23]. While chronic symptoms such as dyspnea and cough reflect the fibrotic component, the emergence of extrapulmonary manifestations, including glomerulonephritis and DAH, may indicate progression toward systemic involvement [5,8,12,13,17,22,23].

Importantly, although UIP with MPO-ANCA positivity may initially present as a lung-dominant phenotype, the most common clinical manifestations of MPO-ANCA-associated vasculitis in broader practice are renal, frequently occurring within a pulmonary-renal syndrome [5,8,12,13]. This highlights the potential for specialty-related diagnostic bias, in which pulmonary cohorts may overrepresent fibrotic presentations [10,11,21].

DAH represents a severe complication associated with increased mortality and may present with non-specific features that can overlap with acute exacerbations of interstitial lung disease, potentially delaying recognition [12,13,17,23]. These observations underscore the importance of maintaining a high index of suspicion for vasculitic progression in MPO-ANCA-positive patients, particularly in the presence of atypical clinical deterioration or new systemic findings [10,13,17,23].

The identification of CPFE as a frequent and clinically relevant phenotype further underscores the complexity of MPO-ANCA-associated UIP [8,10-12]. This overlap syndrome, characterized by relatively preserved lung volumes but markedly reduced diffusing capacity, reflects the coexistence of parenchymal and vascular injury and has been associated with an increased risk of pulmonary hypertension and mortality [8,10-12].

The presence of CPFE may complicate functional assessment, as conventional spirometric parameters can underestimate disease severity despite significant impairment in gas exchange [10-12]. Moreover, this phenotype supports the concept of a multifactorial disease process in which chronic injury, immune dysregulation, and vascular remodeling may interact [3,10,21].

From a diagnostic perspective, the integration of serological and radiological data is essential [2,3,10,21]. While HRCT reliably identifies a UIP pattern, it lacks specificity for distinguishing idiopathic from autoimmune-associated disease [2,3,11]. In this context, MPO-ANCA testing represents a valuable tool for phenotypic characterization, particularly in patients with atypical clinical features, inflammatory laboratory abnormalities, or progressive disease [3,10,21].

Importantly, reliance on imaging alone may lead to misclassification of a subset of patients who may subsequently develop systemic vasculitis [10,19,21]. In most cases, the combination of HRCT findings and serological evaluation is sufficient for diagnostic assessment, and lung biopsy is rarely required, being reserved for cases with persistent diagnostic uncertainty [2,3,10].

These considerations support a more comprehensive diagnostic approach that integrates clinical, serological, and radiological data, along with structured longitudinal follow-up to detect early signs of systemic progression [10,14,21].

Therapeutic decision-making remains one of the most challenging aspects of this entity, as current strategies must balance the relative contributions of fibrotic and inflammatory mechanisms [3,5,11,14,21]. Although established international guidelines exist for the management of ANCA-associated vasculitis and fibrotic interstitial lung disease as separate conditions, no specific recommendations currently address this combined phenotype [2,3,21].

In clinical practice, immunosuppressive therapy is generally indicated in the presence of active vasculitis or organ-threatening manifestations such as DAH, although its impact on fibrotic progression appears variable [3,5,8,11,14,18]. Conversely, antifibrotic agents target the dominant fibrotic pathway but do not address the underlying autoimmune process [2,3,14].

This therapeutic dichotomy supports a phenotype-driven approach, in which treatment is individualized according to the predominant disease component [3,5,11,14,21]. However, the lack of comparative studies and the limited quality of available evidence highlight the need for cautious clinical judgment and multidisciplinary management [3,21].

Prognostically, MPO-ANCA-associated UIP is linked to unfavorable outcomes, largely driven by the extent and progression of fibrosis, as well as the development of systemic complications [5,7,8,11,16,20,22,23]. Several factors, including advanced age, a UIP pattern on imaging, decline in pulmonary function, and the presence of CPFE or vasculitic manifestations, have been associated with worse survival across multiple studies [7,8,11,16,20-23]. These observations underscore the importance of early risk stratification and close clinical monitoring, with the aim of anticipating both chronic disease progression and acute systemic events [10,14,17,21].

In this context, longitudinal surveillance represents a central component of patient management [10,14,21]. Baseline MPO-ANCA testing, followed by periodic reassessment of serological markers, renal function, and overall clinical status, may facilitate the early identification of disease evolution [3,10,14,21]. The recognition of subtle findings, such as microscopic hematuria or unexplained anemia, may indicate early systemic involvement and should prompt further evaluation and timely adjustment of therapy [8,12,13,17]. This structured approach extends beyond conventional IPF follow-up strategies and supports a more dynamic, phenotype-oriented, and multidisciplinary model of care [3,10,21,24].

Proposed Clinical Approach

Based on the evidence summarized in Tables 1-3, a practical clinical approach to MPO-ANCA-positive interstitial lung disease should begin with confirmation of the radiological pattern on HRCT, particularly definite or probable UIP. In patients with fibrotic interstitial lung disease and atypical clinical, laboratory, or radiological features, MPO-ANCA testing should be considered to identify a potential autoimmune-associated phenotype. Once MPO-ANCA positivity is detected, clinicians should assess for systemic vasculitic involvement, including renal dysfunction, hematuria, constitutional symptoms, anemia, and diffuse alveolar hemorrhage. Patients without overt vasculitis should undergo structured longitudinal surveillance with pulmonary function testing, serial serological and renal assessment, and follow-up imaging. Therapeutic decisions should be guided by the predominant phenotype: antifibrotic therapy may be considered in progressive fibrotic-predominant disease, whereas immunosuppressive therapy should be prioritized in patients with active systemic vasculitis or organ-threatening manifestations. Multidisciplinary evaluation involving pulmonology, rheumatology, nephrology, and radiology remains essential.

Despite growing recognition of this entity, the current body of evidence remains limited by the predominance of retrospective studies, small sample sizes, and substantial heterogeneity in diagnostic definitions and outcome reporting [3,21]. The relative scarcity of prospective cohorts and the absence of randomized clinical trials restrict the ability to establish evidence-based management strategies and to more precisely characterize the natural history of the disease [3,21].

As a result, clinical decision-making often relies on extrapolation from related conditions and expert consensus [3,21]. These limitations highlight the need for well-designed prospective and multicenter studies to improve phenotypic characterization and inform more robust diagnostic and therapeutic frameworks [3,21].

In summary, MPO-ANCA-associated UIP can be understood as an evolving clinical phenotype that challenges the traditional boundaries between fibrotic lung disease and systemic vasculitis [3,10,21]. Recognizing this entity has important implications for diagnosis, monitoring, and treatment, as it supports a shift from a static, fibrosis-centered model toward a dynamic, phenotype-driven approach [3,10,21].

Future research should focus on clarifying underlying disease mechanisms, refining classification criteria, and developing therapeutic strategies that address both the fibrotic and autoimmune components of this complex condition [3,21].

## Conclusions

MPO-ANCA-associated UIP can be understood as an evolving clinical phenotype that extends beyond the conventional framework of IPF, reflecting the interplay between fibrotic lung disease and systemic autoimmunity. Clinically, this phenotype is characterized by a dynamic disease course combining progressive fibrosis with the potential for autoimmune activation and systemic complications, including DAH and rapidly progressive glomerulonephritis. These features support the importance of incorporating serological evaluation, structured longitudinal monitoring, and multidisciplinary assessment into patient care to facilitate early recognition of systemic involvement. Improved recognition of this phenotype may enhance risk stratification and support more timely, individualized management strategies.
